# Stability of orthodontic treatment and dental extractions

**DOI:** 10.1590/2177-6709.22.3.009-010.edt

**Published:** 2017

**Authors:** David Normando, Guilherme Janson

**Affiliations:** 1 Adjunct Professor of Orthodontics, School of Dentistry, Universidade Federal do Pará (UFPA). Coordinator of the Graduate program in Dentistry at UFPA and the Specialization Course in Orthodontics at ABO-Pará.; 2 Full Professor of Orthodontics at Bauru School of Dentistry, Universidade de São Paulo (FOB-USP).

*“Success is the ability to go from failure to failure without losing your enthusiasm”*

Winston Churchill, British politician and writer.

Today’s orthodontics has substantially reduced the need for extractions. One of the reasons for this change in approach is based on the findings of studies published in the early 1980s by researchers at the University of Washington, who reported similar rates of teeth alignment relapse in cases treated with or without premolar extractions.^1^ ^,^ ^2^ Thus, it was reported that the decision to extract teeth would not improve the chances of increased long term orthodontic treatment stability. However, Little et al^1^ ^,^ ^2^ focused their studies on the changes that occurred in teeth alignment. If one glimpses beyond crowding, an analysis of the literature will undoubtedly reveal that this assumption may be wrong, and the reverberating truth may in the future damage our ears, as it will certainly damage the patients’ teeth.

Regarding the stability of sagittal correction, more specifically in the treatment of Class II malocclusion, there used to be a dogma, still recurrent in Orthodontics, that the molars should be finished in a Class I relationship to provide greater treatment stability.^3^ Nevertheless, this assertion finds no support when treatment stability is analyzed comparatively, i.e., between protocols involving extractions of two maxillary premolars and nonextraction protocols.^4^ A recent systematic review indicated as inconclusive whether or not stability would be achieved to a greater or lesser degree if a Class II treatment were performed with or without extractions.^5^ On the other hand, there seems to be evidence, albeit relatively scant, indicating that cases with Class III malocclusion treated compensatorily with extractions of lower teeth are more stable than nonextraction cases.^6^ ^,^ ^7^

Even if extractions appear to lessen the risk of relapse of compensatory orthodontic treatment of Class III malocclusion, the same results have been yielded by studies examining stability of non-surgical treatment of anterior open bite. Clinical studies have revealed that stability seems to improve when treatment in the permanent dentition is performed with extractions, be it in adolescents^8^ or in adults.^9^ It has been speculated that stability is improved in extraction treatments precisely because two mechanisms help in closing the anterior open bite, i.e., the “drawbridge principle,” and the mesialization of posterior teeth.^8^

But why should we broach this subject again? Thanks to the advent and popularization of skeletal anchorage there has been a vast increase in treatment approaches whereby sagittal skeletal errors are compensated by en mass distalization of the maxillary teeth in Class II cases, or the mandibular teeth, in Class III cases. Furthermore, the option of intruding the posterior teeth in cases of anterior open bite with the help of skeletal anchorage devices has been increasingly gaining momentum. These treatment protocols boast the unique advantage of reducing the indication of extractions. Conversely, if the prospect of a lower chance of relapse of sagittal and vertical changes (anterior open bite) in extraction cases is true, it is highly likely that we are raising the risk of relapse with the aid of skeletal anchorage, in nonextraction cases.

At this point, when opting for such a treatment protocol, it would be wise to inform the patient that we are embarking on a treatment with a higher risk of relapse. At least for now, while we wait for a scientific answer.

David Normando, Guilherme Janson
